# An Extremely Rare Case of Disseminated Peritoneal Leiomyomatosis with a Pelvic Leiomyosarcoma and Omental Metastasis after Laparoscopic Morcellation: Systematic Review of the Literature

**DOI:** 10.3390/diagnostics12123219

**Published:** 2022-12-19

**Authors:** Antonella Vimercati, Carla Mariaflavia Santarsiero, Angela Esposito, Carmela Putino, Antonio Malvasi, Gianluca Raffaello Damiani, Antonio Simone Laganà, Amerigo Vitagliano, Marco Marinaccio, Leonardo Resta, Ettore Cicinelli, Gerardo Cazzato, Eliano Cascardi, Miriam Dellino

**Affiliations:** 1 Obstetrics and Gynecology Unit, Department of Biomedical Sciences and Human Oncology, University of Bari “Aldo Moro”, Piazza Giulio Cesare 11, 70124 Bari, Italy; 2 Unit of Gynecologic Oncology, ARNAS “Civico—Di Cristina—Benfratelli”, Department of Health Promotion, Mother and Child Care, Internal Medicine and Medical Specialties (PROMISE), University of Palermo, 90127 Palermo, Italy; 3 Department of Emergency and Organ Transplantation, Pathology Section, University of Bari “Aldo Moro”, Piazza Giulio Cesare 11, 70124 Bari, Italy; 4 Department of Medical Sciences, University of Turin, 10124 Turin, Italy; 5 Pathology Unit, FPO-IRCCS Candiolo Cancer Institute, 10060 Candiolo, Italy

**Keywords:** disseminated peritoneal leiomyomatosis, pelvic leiomyosarcoma, omental metastasis, laparoscopic morcellation

## Abstract

Minimally invasive treatment of uterine fibroids usually requires a power morcellation, which could be associated with several complications. A rare sequela is disseminated peritoneal leiomyomatosis. Indeed, recurrence or metastasis in these cases could be attributed to iatrogenic or under-evaluation of primary tumors, although a subset of cases is a sporadic sample of biological progression. We present an extremely rare case of a patient who underwent laparoscopic morcellation and after 12 years developed a pelvic leiomyosarcoma with two omental metastases, disseminated peritoneal leiomyomatosis with a parasite leiomyoma with bizarre nuclei and a parasite cellular leiomyoma simultaneously. The diagnosis was predicted preoperatively by an expert sonographer who recognized the ultrasound characteristics of uterine sarcoma and the localization of some of the masses, so the patient was referred to the gynaecological oncologists who could appropriately treat her. We present here a case report and a systematic review that could be a useful tool for further discussion and future clinical practice guidelines.

## 1. Introduction

Uterine leiomyoma is the most common benign tumor in women. Its prevalence is approximately 70–80% in women by the age of 50 [1]. Surgical treatment of uterine myoma, if indicated, could be performed by traditional laparotomy, vaginally or with minimally invasive surgery. Laparoscopy often requires a power morcellation of the myoma to remove it through a small excision [2]. However, laparoscopic power morcellation may determine the spreading of an unsuspected leiomyosarcoma into the peritoneal cavity, which is associated with a worse prognosis [3,4]. Other possible consequences of morcellation are the intraperitoneal implants of endometriosis (1.4%), adenomyosis (0.57%) and parasitic leiomyomas. A very rare sequela is disseminated peritoneal leiomyomatosis (DPL), a condition characterized by the presence of multiple smooth muscle implants on the abdominal and pelvic peritoneum and on the omentum [3,5]. DPL is considered a benign condition, in fact only a few cases of malignant transformation of DPL have been reported in the literature [6,7,8,9,10,11,12,13,14,15,16]. We present an extremely rare case of a patient who underwent laparoscopic morcellation and after 12 years developed a pelvic leiomyosarcoma with two omental metastases, pelvic endometriosis, disseminated peritoneal leiomyomatosis with a parasite leiomyoma with bizarre nuclei and a parasite cellular leiomyoma simultaneously.

## 2. Case Presentations

**Figure 1:**
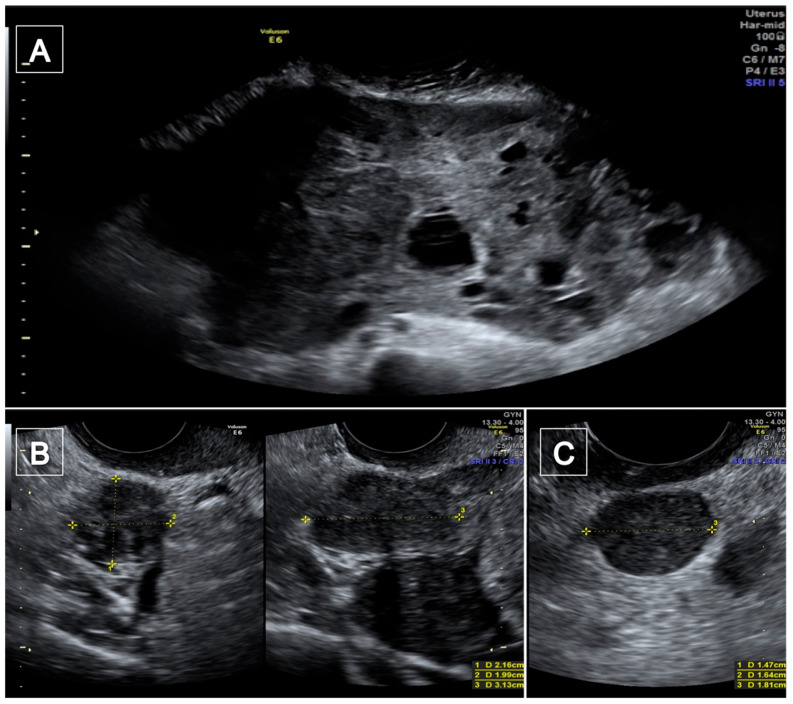
The trans-abdominal ultrasound examination. A voluminous mass with irregular margins and a heterogeneous echotexture for the presence of some cystic areas with anechoic content (**A**); two masses behind the uterus with regular margins, inhomogeneous echotexture and with acoustic shadows (**B**); a lesion of 14 × 16 × 18 mm with regular margins (**C**).

A 47-year-old woman (para 2/0/0/2) presented to our hospital in March 2021 with lower abdominal pain, not responsive to medical therapy. She had regular menses and she had previously taken oral contraceptives (gestodene + etinyl estradiol) for 12 years until the age of 30. Her cousin had died from a uterine leiomyosarcoma at 49 years old. In 2008 she underwent a laparoscopic myomectomy for a rapidly growing uterine intramural-subserosal myoma, and power morcellation was performed. Histological examination was positive for uterine leiomyoma. At admission, the physical examination showed abdominal distension caused by a voluminous hard palpable mass, which ended four fingers above the transverse umbilical line. The computed tomography scan of the abdomen and of the pelvis, performed in the emergency department, revealed a voluminous inhomogeneous solid mass of 24 × 13 × 23 cm in size, referable to a right adnexal mass; the appearances of the uterus and the left adnexa at CT scan were regular. The tumor markers (Ca125, Ca19.9, CEA, Ca15.3, AFP) were negative and the LDH levels were 575 U/L. The transvaginal and transabdominal ultrasound examination showed a normal anteverted uterus, of 73 mm in size, with irregular margins and an inhomogeneous echo pattern. The endometrial echo pattern was secretive, with an endometrial thickness of 7 mm. The ovaries were both regular in size, shape and echotexture. A voluminous mass of 253 × 151 × 235 mm in size occupied the pelvis and the abdomen. The mass showed irregular margins and an inhomogeneous echotexture for the presence of some cystic areas with anechoic content. The lesion showed stripes and it appeared richly vascularized at color Doppler examination (Figure 1A). The large mass seemed to be a myometrial lesion with signs of malignancy, but it showed no vascular connection with the uterine walls. Furthermore, some additional masses without vascular connection with the uterine walls were seen in the pelvis: one left lateral-sided mass of 43 ×38 × 39 mm and two anterior masses of 21 × 19 × 31 mm and 15 × 12 × 20 mm with regular margins, inhomogeneous echotexture, acoustic shadows, and without vascularization at color Doppler examination (Figure 1B), and a right-sided lesion of 14 × 16 × 18 mm, with regular margins, inhomogeneous echotexture, without acoustic shadows and moderately vascularized at color Doppler examination (Figure 1C).

**Figure 2:**
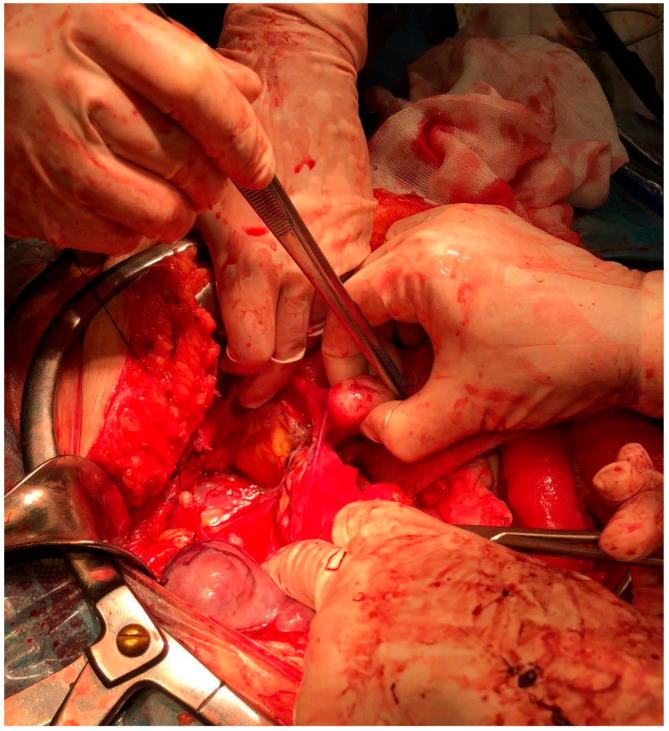
A picture from the operating room after removal of the largest mass. From this shot you can see the numerous rounded formations and how they relate to the other abdominal structures.

No pelvic free fluid and no ascites were seen. The patient underwent a laparotomic surgery. The exploration of the abdomen and of the pelvis confirmed the presence of a large lobulated mass of about 30 cm, which presented necrotic, colliquate and hemorrhagic areas (Figure 2).

It was tenaciously adherent to the prevesical peritoneum, to the small intestine, to the left colon and to the sigma. The uterus appeared irregular in shape. The adnexa were both regular in size and shape. Further nodular lesions on the abdominal and pelvic peritoneum were detected. Two omental lesions were also seen. The patient underwent a total hysterectomy, a bilateral salpingo-oophorectomy, and an omentectomy, and all the peritoneal nodules were carefully removed. The final pathology of the large abdominal–pelvic mass was positive for leiomyosarcoma of high grade, with extensive necrosis, widespread severe cytokaryological atypia, and a high mitotic index (>20/10 HPF), ki-67 = 70%. A small peritoneal lesion was a focus of endometriosis. The mass of the right pelvic peritoneum had cellulate aspects with ki-67 = 10%, and it was defined as a cellulate leiomyoma. A lesion of the posterior wall of 6 cm was a leiomyoma with bizarre nuclei. All the other four isolated masses were myomas. The omental lesions were both positive for leiomyosarcoma metastases. The post-operative course was uneventful and free from surgical complications, and the patient was discharged on the ninth post-operative day, with a total regression of the initial symptoms.

## 3. Methods

**Figure 3:**
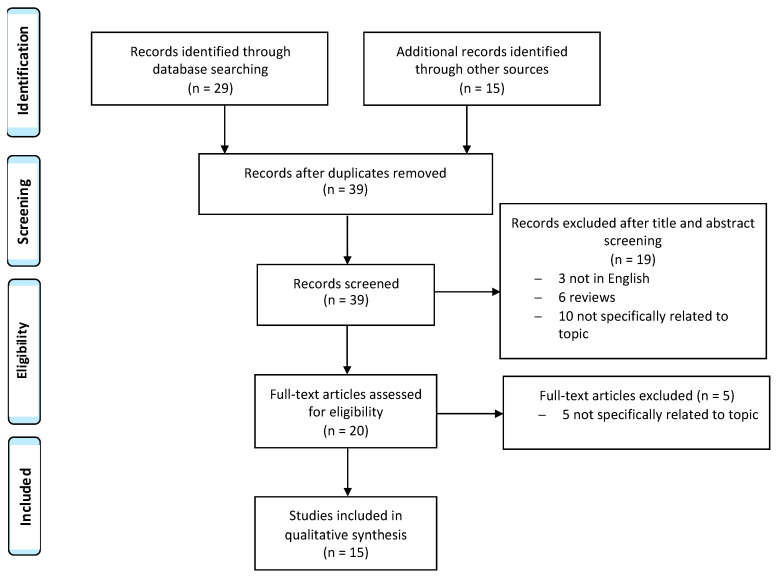
Study flow diagram: PRISMA flow diagram of identification, screening and inclusion of articles. Systematic literature reviews, were selected with standard methods to be briefly presented in the article. The search identified 15 studies with the desired characteristics.

A systematic review of DPL with malignant transformation reports was performed through a literature search in the following electronic databases: PubMed, the Cochrane Library, and Web of Science. The article research was performed in agreement with preferred reporting items for systematic reviews and meta-analyses (PRISMA) The following search terms were used: “disseminated peritoneal leiomyomatosis, malignant transformation, pelvic leiomyosarcoma, sarcoma, omental metastasis and laparoscopic morcellation”. We considered particular articles, case series and case reports published in English (Figure 3). No restrictions on the year of publication were applied. A manual search of the reference list of each study was performed to avoid missing relevant publications. Titles and abstracts of the eligible articles were independently reviewed by two authors (M.D and C.M.S.). Duplicates were removed. The full texts of potentially suitable studies were independently assessed for eligibility by the two authors. Any discordance between the two sides was solved through discussion with two senior reviewers (A.V. and E.C.).

## 4. Discussion

Most mesenchymal cancers that occur in the uterus are smooth muscle cancers. The majority are classified as leiomyomas, and subtypes of leiomyoma have been classified according to the World Health Organization (WHO) classification (4th edition). Kurman RJ, Carcangiu ML, Herrington C, Young RH, editors. WHO Classification of Tumours of Female Reproductive Organs. Lyon: IARC; 2014. Conservative follow-up in the absence of symptoms and procedures of myomectomy or hysterectomy can be completely curative in cases where surgical treatment is selected. However, although such tumors are histologically considered benign after treatment, some cases still show local recurrences or distant metastases. Only a few cases of DPL with malignant transformations are described in the literature (Table 1). The electronic search based on our pre-defined key search item identified 44 records, which became 39 after the removal of duplicates. After title and abstract screening, 19 records were excluded and 20 full-text articles were assessed for eligibility. Finally, 15 studies dating from 1986 to 2022 were included in the qualitative synthesis. The studies available were: one case series of 5 cases [17] and 14 case reports.

We collected these previous cases and summarized their clinical features (patient’s age, the number and the localization of the myomas, the histotype of the sarcoma and eventual metastases) and the pre-operative examinations performed. Moreover, we reported if the diagnosis of malignancy was made or suspected preoperatively or only after surgery. The patients’ age ranged from 20 [20] to 72 years [16], with a mean age of 41.2 years. The disseminated myomas were mainly localized in the pelvis, but retroperitoneal lesions were also detected [6,7]. Some authors described leiomyomatous nodules of the omentum [7,8,11,15] and of the intestinal serosa [11,12,13,14,16,17,19]. Two cases of leiomyomatous nodule of the liver [15,17] were reported, as well as two cases of subdiaphragmatic peritoneum lesion [9,17]. Wen et al. detected a mass involving the right ureter mimicking metastatic urinary tract cancer [16]. Almost all malignant transformations were leiomyosarcomas, and two cases of pulmonary and hepatic metastases were reported [6,15]. Rosati et al. identified a spindle cell sarcoma, the mass described by Zyla et al. was an endometrial stromal sarcoma [7], while Rubin et al. found a small spindle cell sarcoma with diffuse bone metastases [18] and Wen et al. identified a myxoid leiomyosarcoma. In one case [18], the masses were accidentally discovered at a cesarean section. In 10.5% of cases, any pre-operative examination was made. In 21% of cases only, an ultrasound examination was performed, while in all the other cases, patients underwent abdominal and pelvic CT or MRI scan. Although several diagnostic techniques were performed, only in a few cases was the diagnosis made before surgery [6,8,10,19,21]. A recent study reporting approximately 62 cases with a histopathological diagnosis of leiomyoma and the presence of subsequent recurrences or metastases revealed the heterogeneous nature of this category of tumors [22,23]. The causes of recurrence and metastasis are multiple in this iatrogenic etiology. In fact, in about 75.8% of recurrent cases there was a history of laparoscopic myomectomy, and in 79% of these a morcellator without a bursa was used [24]. Recurrent tumors have been identified predominantly in regions close to intraoperative fields, and the median time to recurrence of these tumors reported in the literature is on average 51.5 months [22,25]. These data suggest that a subgroup of metastatic leiomyoma is iatrogenic due to the use of a morcellator without a bursa. Therefore, the U.S. Food and Drug Administration (FDA) recommends the use of bags in case of procedures involving slaughterers [22,26]. On the other hand, hormone therapy is considered the treatment of first choice if the tumor can be determined as benign [10,11,27]. However, there are difficulties in histological evaluation and application of the Stanford criteria. Currently, the Stanford criteria, which have been incorporated into the WHO classifications, are used to establish the histopathological diagnosis of leiomyosarcomas.

**Figure 4:**
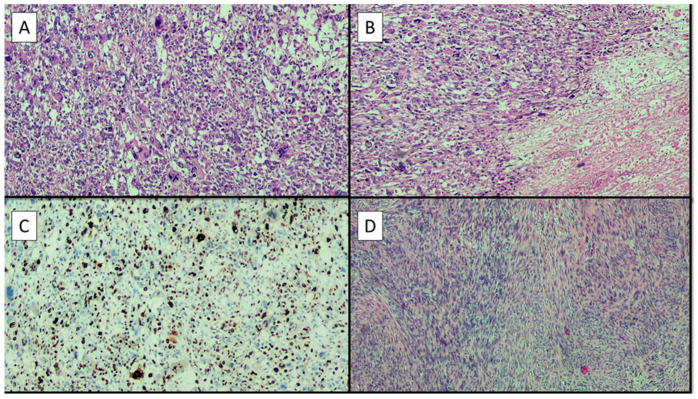
A collage of histological images of the case presented: (**A**) Histological preparation showing a proliferation of high-grade spindle and/or pleomorphic cells with eosinophilic cytoplasm, often forming interlacing but disorganized fascicles. Note diffuse pleomorphism of the nuclei (hematoxylin–eosin, original magnification 4×); (**B**) in this histological preparation it is possible to appreciate on the right the presence of tumoral necrosis. (hematoxylin–eosin, original magnification 4×); (**C**) immunohistochemical preparation with antibody anti-Ki67 antigen: note the high presence of neoplastic cells immunolabeling in brown (immunohistochemistry, original magnification 4×); (**D**) histological picture of a leiomyoma of this patient. Note the almost total absence of pleomorphism, nuclear atypia and necrosis. (hematoxylin–eosin, original magnification 4×).

There are several criteria through which the pathologist makes the diagnosis of leiomyoma or leiomyosarcomas. Macroscopic evaluation is undoubtedly essential. For this reason, where possible, hysterectomy or myomectomy is always preferable to morcellation, since in the first two cases, a complete and oriented evaluation of the anatomical piece is always possible. Moreover, as demonstrated in the literature, transvaginal extraction in the bag can also be considered a safe option for the recovery of surgical samples after laparoscopic myomectomy, for which rare and transient complications (0.6%) such as vaginal bleeding, and 0.3% vaginal pain are found [28,29]. Intramural formations rather than submucosal or subserosal formations are thus easily identifiable, as is their correct number. Despite being without a capsule, leiomyomas generally appear macroscopically with well-circumscribed and rounded margins, with the typical fasciculate-like appearance to the cut that characterizes a lesion of increased consistency, with a white-gray complexion, rarely presenting even coarse calcifications [30]. Bleeding areas may be present. Conversely, leiomyosarcoma tends to have a blurred outline whose limit may not be easily identified and may present areas of necrotic degeneration [30]. For this reason, in uterine stromal lesions a large sampling is always recommended in order to identify myxoid or degenerative areas [30]. In cases of leiomyoma, a simple of hematoxylin and eosin slide, evaluated at low magnification, allows confirmation of the presence of well-demarcated margins, while at higher magnification the cytomorphological details confirm the benign nature of the lesion (Figure 4).

In fact, tumor cells do not present an important pleomorphism, usually being all fusocellular/spindle, monomorphic, with an eosinophilic cytoplasm and elongated cigar-shaped nucleus almost always devoid of nucleoli; mitotitis figures are practically absent or very rare, as are tumor necrosis or other types of cellular degeneration [31,32]. Although rare, bizarre forms of leiomyoma are still described [33,34,35,36,37] which still maintain a low mitotic index. The sarcomatous component, on the other hand, presents an increased stromal cellularity which is typically accompanied by a marked cellular pleomorphism due to neoplastic cells with marked inversion of the nucleus/cytoplasm ratio, multinucleolated cells and more numerous mitotic figures (>10 mitoses/10 HPF of 0.55 mm in diameter) often also atypical. Necrosis may not be identifiable but where present it remains a very useful finding for the differential diagnosis. The presence of two or more histological criteria, previously described, allow the pathologist to confirm the benign, malignant or uncertain potential of malignancy (STUMP) nature of the neoplasm. This new entity deserves particular attention as it may present bridging characteristics between the classic leiomyoma and its malignant version (leiomyosarcoma). In fact, STUMP in radiographic investigations usually appears as a well-circumscribed mass, with little blood circulation, which, however, due to intratumoral necrosis, can be difficult to interpret on magnetic resonance imaging [38]. In addition, histologically, the evaluation of an expert pathologist is recommended, given the different cytological forms it can take: spindle, myxoid and epithelioid STUMP. These women must be included in very stringent surveillance protocols for at least the first 5 years after diagnosis [39], by virtue of the fact that up to 30% of cases can recur [39]. This is more susceptible in the epithelioid and myxoid forms [40]. Furthermore, the prognosis to-date remains uncertain in most cases and immunohistochemical markers do not seem to have a prognostic role so far, while genetic alterations such as loss of ATRX or DAXX expression or of chromosome 13 are associated with a worse prognosis [41,42,43,44]. Immunohistochemistry represents a valid support where these neoplasms have different souls and therefore deserve diagnostic investigations. In cases of small biopsies before surgery, as well as for other fusocellulated tumors, it is also necessary to investigate the lesion with a pancytokeratin to exclude the epithelial nature of the neoplasm [45,46]. Usually this family of neoplasms are positive desmin, caldesmone and actin smooth muscle [47]. Markers of melanocytic derivation such as HMB45 and MelanA [48,49,50] can instead direct towards the diagnosis of pecoma [51,52], just as p16, which is notoriously negative in indolent forms, and strongly expressed in bizarre histological forms [53].

In cases of leiomyosarcoma, there have also been several genetic mutations among which we most frequently find TP53, ATRX and MED12 [44], in addition to NR4A3-PGR fusions or PGR rearrangements described in about one third of cases with a rich epithelioid component [54]. A similar percentage has been reported for PLAG1 rearrangement in high-grade forms with myxoid aspects [55].

Patients with uterine leiomyosarcomas have a poor prognosis, considering they tend to be very aggressive and often recur. The literature reports a percentage that can even touch 70% with a maximum survival of 70% at 5 years in stages I and II, while patients in more advanced stages see this percentage reduced up to just 10 months from diagnosis [56,57]. The main predictors of this trend are lymphovascular involvement, high mitotic index, high-grade nuclear atypia and disease stage but also tumor size and regional/distant metastases [58,59]. Regardless of histotype, in stage I–II operated patients, adjuvant radiotherapy does not reduce overall survival (OS) since it tends to decrease loco-regional but not distant metastasis [60,61]. Adjuvant chemotherapy, on the other hand, is currently a rather debated topic. Indeed, in stages I and II a retrospective analysis of 2732 women with non-metastatic leiomyosarcoma showed that the use of adjuvant chemotherapy had no impact on OS [62]. Similar data emerged from another study comparing a combined regimen of adriamycin+ifosfamide+cisplatin followed by radiotherapy versus radiotherapy alone [63]. This study did not show an improvement in OS at 3 years in the two groups [63]. In contrast, the combination of gemcitabine and docetaxel showed higher than expected progression-free survival in two studies [64,65]. In stages III and IV, there is no standard therapy and the decision is taken into consideration of the possibility of surgery with complete removal of the mass. Then, patients are offered chemotherapy regimens (anthracycline ± ifosfamide or anthracycline ± dacarbazine or gemcitabine ± docetaxel). However, it should be considered that the studies that analyzed these possibilities almost all had the limit of a small number of cases [64,65]. The same evaluation emerges from some meta-analyses, which demonstrate the advantage of chemotherapy treatments but with the same limitation as the previous data [66,67]. In inoperable patients, the use of chemotherapy has shown encouraging results, also allowing an overall response to gemcitabine and docetaxel [68] or slowing down relapses [69,70].

## 5. Conclusions

Our work confirms the correlation between laparoscopic morcellation of uterine myomas and the onset after several years of some complications such as DPL and endometriosis. Therefore, morcellation with a pouch would be mandatory to avoid iatrogenic recurrence. On the other hand, although a subset of cases has shown rare examples of biological progression, recurrence or metastasis, these can sometimes be related to iatrogenic or undervalued tumors of primary tumors. Therefore, more sophisticated diagnostic criteria for uterine smooth muscle tumors should be applied in a multidisciplinary manner so that diagnostic and clinical information between gynecologists and pathologists can be correlated for optimal patient management, in order to avoid recurrences due to incorrect primary diagnosis. In addition, the present case shows the simultaneous presence of pelvic endometriosis, a large leiomyosarcoma with omental metastases and six multiple myomas with different histotypic characteristics, and was the first case to report malignant DPL degeneration with omental metastases. 

In cases of uterine stromal tumors, after having carefully radiologically investigated this type of tumor, en bloc resection of the mass would always be preferable. Furthermore, in case of suspected sarcoma, the patient must be managed from a diagnostic and surgical point of view in centers with specialized experience and a large number of cases. This would allow patients to be referred to gynecological oncologists who can treat them appropriately.

## Figures and Tables

**Table 1:** Cases of disseminated peritoneal leiomyomatosis with malignant transformation. Abbreviations: CT: computed tomography, MRI: magnetic resonance imaging, ND: not determined, TV: trans-vaginal, * case described in the current manuscript.

Author and Year	Age	Number of DPL	Location	Type of Sarcoma	Pre-Operative Examinations	Pre-Operative Diagnosis
Chiu et al. 2018 [6]	61	3	Retroperitoneal pelvic cavity	high-grade leiomyosarcoma with peritoneal carcinomatosis and pulmonary and hepatic metastases	TV ultrasound CT whole body scan Chest X ray	yes
Rubin et al. 1986 [18]	27	ND	Pelvis	Small spindle cell sarcoma and diffuse bone metastases	No	No, found at cesarean section
Zyla et al. 2015 [7]	26	ND (numerous)	Pelvis Peritoneal cavity Omentum Retroperitoneal space	Low-grade Endometrial stromal sarcoma	No	No
Sharma et al. 2004 [8]	55	ND (multiple)	Omentum Mesentery	Leiomyosarcoma	TV Ultrasound	Yes
Fulcher et al. 1998 [9]	48	ND (more than four)	Pelvis Subdiaphragmatic peritoneum	Moderate-grade Leiomyosarcoma	Renal sonography	No
Lamarca et al. 2011 [10]	37	ND (multiple)	Peritoneal cavity	Leiomyosarcoma	“Imaging techniques”	Yes
Akkersdijk et al. 1990 [11]	25	ND (multiple)	Omentum Colon Small intestine	High-grade Leiomyosarcoma	TV * Ultrasound	No
Raspagliesi et al. 1996 [12]	26	4	Adnex Mesosigmoid Sigmoid serosa	High-grade Leiomyosarcoma	Ultrasound	No
Morizaki et al. 1999 [13]	33	ND (multiple)	Peritoneum Mesentery Descending Colon Pelvis	Leiomyosarcoma and Fibrosarcoma	“Serial examinations”	No
Xu et al. 2019 [14]	47	ND (multiple)	Surface of retroperitoneum sigmoid colon urinary bladder	Leiomyosarcoma	3D TV Ultrasound MRI *	No
Tun et al. 2016 [15]	56	ND (multiple)	Pelvis Peritoneum Omentum Liver	Leiomyosarcoma with lung and liver metastases	“Imaging studies”	No
Syed et al. 2017 [19]	40	ND (multiple)	Peritoneum Recto-uterine pouch Prevesical space Left rectus Abdominis muscle	Leiomyosarcoma	Ultrasound Contrast-enhanced CT scan MRI	Yes
Abulafia et al. 1993 [20]	20	ND (multiple)	Pelvis Omentum	Low grade Leiomyosarcoma	Ultrasound CT scan	No
Rosati et al. 2021 [17]	49	ND (multiple)	Pelvis	Low grade Leiomyosarcoma	All patients underwent CT scan or MRI and abdominal/transvaginal ultrasound	Not available
	36	ND (multiple)	Diaphragm Liver	Low grade Leiomyosarcoma		Not available
	31	ND	Peritoneum	Spindle cell Sarcoma		Not available
	48	ND (multiple)	Abdominal wal Bowel serosa	High grade Leiomyosarcoma		Not available
	46	ND (multiple)	Peritoneum Bowel’s serosa	Low grade Leiomyosarcoma		Not available
Wen et al. 2022 [16]	72	multiple	Rectus Sigmoid colon Bilateral inguinal areas Right ureter	Myxoid leiomyosarcoma	CT scan	No
Vimercati et al. 2022 *	47	6	Pelvis	High-grade Leiomyosarcoma with two omental metastases	TV ultrasound CT scan	Yes

## Data Availability

All data are reported in the text.
